# Mature and progenitor endothelial cells perform angiogenesis also under protease inhibition: the amoeboid angiogenesis

**DOI:** 10.1186/s13046-018-0742-2

**Published:** 2018-04-03

**Authors:** Anastasia Chillà, Francesca Margheri, Alessio Biagioni, Mario Del Rosso, Gabriella Fibbi, Anna Laurenzana

**Affiliations:** Department of Experimental and Clinical Biomedical Sciences “Mario Serio”, Section of Experimental Pathology and Oncology, Viale G.B. Morgagni, 50-50134 Florence, Italy

## Abstract

**Background:**

Controlling vascular growth is a challenging aim for the inhibition of tumor growth and metastasis. The amoeboid and mesenchymal types of invasiveness are two modes of migration interchangeable in cancer cells: the Rac-dependent mesenchymal migration requires the activity of proteases; the Rho-ROCK-dependent amoeboid motility is protease-independent and has never been described in endothelial cells.

**Methods:**

A cocktail of physiologic inhibitors (Ph-C) of serine-proteases, metallo-proteases and cysteine-proteases, mimicking the physiological environment that cells encounter during their migration within the angiogenesis sites was used to induce amoeboid style migration of Endothelial colony forming cells (ECFCs) and mature endothelial cells (ECs). To evaluate the mesenchymal-ameboid transition RhoA and Rac1 activation assays were performed along with immunofluorescence analysis of proteins involved in cytoskeleton organization. Cell invasion was studied in Boyden chambers and Matrigel plug assay for the in vivo angiogenesis.

**Results:**

In the present study we showed in both ECFCs and ECs, a decrease of activated Rac1 and an increase of activated RhoA upon shifting of cells to the amoeboid conditions. In presence of Ph-C inhibitors both cell lines acquired a round morphology and Matrigel invasion was greatly enhanced with respect to that observed in the absence of protease inhibition. We also observed that the urokinase-plasminogen-activator (uPAR) receptor silencing and uPAR-integrin uncoupling with the M25 peptide abolished both mesenchymal and amoeboid angiogenesis of ECFCs and ECs in vitro and in vivo, indicating a role of the uPAR-integrin-actin axis in the regulation of amoeboid angiogenesi**s**. Furthermore, under amoeboid conditions endothelial cells seem to be indifferent to VEGF stimulation, which induces an amoeboid signaling pattern also in mesenchymal conditions.

**Conclusion:**

Here we first provide a data set disclosing that endothelial cells can move and differentiate into vascular structures in vitro and in vivo also in the absence of proteases activity, performing a new type of neovascularization: the “amoeboid angiogenesis”. uPAR is indispensable for ECs and ECFCs to perform an efficient amoeboid angiogenesis. Therefore, uPAR silencing or the block of its integrin-interaction, together with standard treatment against VEGF, could be a possible solution for angiogenesis inhibition.

**Electronic supplementary material:**

The online version of this article (10.1186/s13046-018-0742-2) contains supplementary material, which is available to authorized users.

## Background

Endothelial cells (ECs) form new blood vessels by migration of collective sprouts of cells that maintain cell-cell junctions [1]. Vascular sprouts are guided by a “pathfinder” tip cell that responds to environment guidance cues, thereby determining vascular patterning [2]. Single mature ECs are believed to migrate by mesenchymal type of motility [3]. In 3D matrices, such motility implies an elongated spindle-like shape of the cell body whose translocation requires the formation of actin-rich lamellipodia and filopodia at the leading edge of the EC: this process is driven by the small GTPases of the Rho family, Rac for lamellipodia and CDC42 for filopodia [4]. Both the leading and trailing edges of the EC establish adhesive interactions with the extracellular matrix (ECM), that serve as attachments for the actin stress fibers to generate forces required to translocate the trailing edge in the direction of the cell movement [5]. Mesenchymal motility is characterized by the activity of membrane-associated proteases: integrins give rise to focal adhesions that recruit proteases thus opening a new path to invading tip cells [3, 6].

The protease-independent amoeboid migration (named after the motility of the amoeba *Dictyostelium Discoideum*) is characterized by fast cycles of contraction and expansion of the cell body, which is round or ellipsoid, obtained by contraction of the cortical actin and myosin filaments with the creation of cell “blebs” [7]. This type of movement, observed also in hematopoietic stem cells and certain tumor cell [1, 8], consists in a sort of “crawling” through less dense compartments of the ECM, driven by short-lived weak interaction of the amoeboid cell with the substrate. The enhanced contractility that enables cells that use the amoeboid strategy to squeeze into gaps of the ECM is promoted by the Rho/ROCK signaling pathway [9].

In tumor cells the amoeboid and mesenchymal type of movement are interchangeable, thus defining the mesenchymal-amoeboid transition (MAT) and the amoeboid-mesenchymal transition (AMT) respectively, that represent a rapid response of cancer cells to microenvironment properties [1, 10].

While mature ECs have never been shown to exhibit an amoeboid behavior, endothelial progenitor cells (EPCs) leave bone marrow as amoeboid cells characterized by a roundish shape and a cortical actin cytoskeleton lacking stress fibers [11]. Of course, EPCs need to migrate through the blood vessel basement membrane and through ECM to home to sites where there is the need to form new vessels. At this step integrins provide a “grip” to EPC migration and proteases open the way across anatomical barriers to cells migrating toward the chemotactic source of pro-angiogenic factors [12]. However, this observation is circumstantial and unable to explain plasticity of EPCs in terms of shifting from mesenchymal to amoeboid movement and viceversa.

Here we show that both mature ECs and endothelial colony forming cells (ECFCs), an EPC subpopulation with robust clonal proliferative potential and the ability to form de novo vessels in vivo, are able to migrate by mesenchymal and amoeboid style in vitro and in vivo. For this purpose we have used a mixture of physiologic inhibitors of serine-proteases, metallo-proteases and cysteine-proteases, thus mimicking a possible alternative physiological environment that ECs and ECFCs may encounter during their migration within the angiogenesis sites. As previously shown for malignant melanoma and prostate cancer cells [13], the receptor of the urokinase-plasminogen-activator (uPAR, CD87), is indispensable for ECs and ECFCs to perform an efficient amoeboid angiogenesis, in terms of cell migration and capillary morphogenesis in vitro and in vessel formation within Matrigel plugs in mice.

## Methods

### Cell lines and culture conditions

Endothelial Colony Forming Cells (ECFCs) were isolated from > 50 ml human umbilical cord blood (UCB) of healthy newborns, as described previously [14, 15], after maternal informed consent and in compliance with Italian legislation, and analyzed for the expression of surface antigens (CD45, CD34, CD31, CD105, ULEX, vWF, KDR, uPAR) by flow-cytometry [14]. ECFCs were grown in EGM-2 culture medium (Lonza), supplemented with 10% FBS (Euroclone) onto gelatin coated dishes. Human microvascular endothelial cells (HMVEC) were purchased from Lonza and were grown in the same conditions of ECFCs.

### In vitro capillary morphogenesis

In vitro capillary morphogenesis was performed as described [14, 15] in tissue culture wells coated with Matrigel (BD Biosciences). ECFCs or HMVECs were plated (18 × 10^3^ /well) in EGM-2 medium, supplemented with 2% FCS and incubated at 37 °C-5% CO2. Results were quantified at 6 h by measuring the branching points of capillary projections. Six to nine photographic fields from three plates were scanned for each point. Results were expressed as % increase/decrease of branching points/field ± SD with respect to control fixed at 100%.

Speed of capillary structures formation was measured by time-lapse capillary morphogenesis assay (Additional file 1).

### 3D-invasion assay with Boyden chambers

Invasion was studied in Boyden chambers in which the upper and lower wells were separated by 8 μm–pore size polycarbonate filters coated with Matrigel (BD Biosciences), as previously described [13]. For details, see (Additional file 1).

### Induction of the amoeboid phenotype and cell viability assay

Protease-indipendent angiogenic properties and invasion were evaluated by in vitro capillary morphogenesis and 3D-Boyden chamber assays, as described above, with Matrigel coating in the presence of a physiological protease inhibitor cocktail, consisting of α2-antiplasmin (plasmin inhibitor; 5 μg/ml), Cystatin (cysteine protease inhibitor; 5 μM), PAI 1 (plasminogen activator inhibitor; 10 ng/ml), TIMP1, TIMP2 and TIMP3 (metallo-protease inhibitors; 0,5 μg/ml each one) purchased by Abcam. A completely artificial protease inhibitor cocktail (composed by Ilomastat, leupeptin, pepstatin A, E-64 and aprotinin; Sigma Aldrich) used in a previous study [13] was also used. Concentrations used were selected according to the manufacturer’s instructions and literature [16, 17]. Protease inhibitor cocktails were added to un-polymerized Matrigel solution on the upper surface of the porous filter. To induce the amoeboid phenotype, cells were treated overnight with the protease inhibitor cocktails at the same concentrations used in the invasion assay. Cell viability upon protease inhibitors treatment was evaluated by Trypan blue dye (Sigma) exclusion assay.

### Collagen degradation assay

ECFC and HMVEC cell suspensions were co-polymerized with Matrigel containing 2% FITC-labeled collagen monomers (Molecular Probes). Digestion was allowed for 40 h at 37 °C and solid-phase Matrigel containing the cells was pelleted, whereas FITC released into the supernatant was analyzed by spectrofluorometry. One hundred percent values were obtained by complete collagenase digestion of cell-free Matrigel lattices. Background fluorescence was obtained by pelleting non-digested cell-free FITC-collagen-enriched Matrigel layers.

### RhoA and Rac1 activity assay

Cells from different experimental conditions (control, physiological protease inhibitor cocktail) were lysed in radio-immunoprecipitation assay buffer, the lysates were clarified by centrifugation, and RhoA GTP or Rac1 GTP was quantified. Briefly, lysates were incubated with 10 μg rhotekin–glutathione S-transferase (GST) fusion protein (Millipore) or p21-activated kinase-GST fusion protein, both absorbed on glutathione–Sepharose beads for 1 h at 4 °C. Ratios between activated (GTP-bound) RhoA and Rac1 adsorbed to the beads were quantified by Western blot densitometry.

### Western blotting

Specific electrophoretic conditions and the source of used antibodies are reported Additional file 1.

### Semiquantitative reverse transcription–polymerase chain reaction (PCR) analysis

Total RNA preparation and reverse transcription were performed as previously reported. The levels of messenger RNA for the integrin chains were determined by an internal-based semiquantitative RT-PCR, using procedures and primers previously described [18].

### Treatment of cells with M25 peptide

Inhibition of uPAR-integrin interaction was obtained with the M25 peptide, previously identified in a phage display library [19], able to uncouple uPAR interaction with integrin α-chain. The peptide was produced in collaboration with PRIMM srl, Milan, Italy. In the β-propeller model of α-chain folding, the sequence of this peptide (STYHHLSLGYMYTLN) spans an exposed loop on the ligand-binding surface of α-chain, thus impairing integrin α chain-uPAR interaction. In cell culture both M25 and scramble-M25 (sM25) were used at 50 μM for 15 h at 37 °C.

### Co-immunoprecipitation

For co-immunoprecipitation, ECFCs and HMVECs were plated at 500 × 10^3^ cells/100 mm dish in complete medium. One of two dishes for each cell line was treated with M25 peptide at 50 μM for 15 h. After two washes in ice-cold PBS, cells were lysed on ice with Ripa buffer, centrifuged (15,000 rpm, 15 min) and the supernatant was used for co-immunoprecipitation. For details, see Additional file 1.

### Immunofluorescence analysis

Immunofluorescence and confocal microscopy were performed as previously described [18]. For details, see Additional file 1.

### siRNA uPAR knock-down and quantitative real-time PCR analysis

See details under Additional file 1.

### In vivo Matrigel plug assay

All procedures involving animals were performed in accordance with the ethical standards and according to the Declaration of Helsinki and to national guidelines approved by the ethical committee of Animal Welfare Office of Italian Health Ministry and conformed to the legal mandates and Italian guidelines for the care and maintenance of laboratory animals.

Two groups of 8 and 10 four-week-old male SCID beige mice (two for each experimental condition) were purchased from Charles River. The Matrigel plug assay was used to study a possible role of the amoeboid movement in vivo as previously described [14]. VEGFA (10 ng/ml), was added to unpolymerized Matrigel at 4 °C at a final volume of 0.6 ml. Protease inhibitors were added to unpolymerized Matrigel at the same concentrations used for in vitro assays. Heparin (50 U/ml) was added to each solution. To study the role of mice vessels, the Matrigel suspension was carefully injected subcutaneously into both flanks of mice using a cold syringe. As the Matrigel warms to body temperature, it polymerizes to a solid gel, which then becomes vascularized within five days in response to the angiogenic substance. The extent of vascularization was quantified by measuring the hemoglobin content of the recovered plugs. Groups of four pellets were injected for each treatment. The eight animals were subdivided as follows: two controls (Matrigel alone); two animals injected with a Matrigel plug containing VEGF-A; two injected with Matrigel plus the physiologic protease-inhibitor cocktail; and two with Matrigel, the physiologic protease-inhibitor cocktail plus VEGFA. The reagents for all treatments were added to the Matrigel solution prior to injection. Five days after injection, the pellets were removed, minced and diluted in water to measure the hemoglobin content with a Drabkin reagent kit (Sigma). Vascularization was evaluated by sight taking a representative photograph of individual Matrigel plugs recovered at autopsy for the corresponding condition. Samples were also fixed in formalin, embedded in paraffin for histological analysis and stained with hematoxylin-eosin.

The second group of 10 mice was used to verify the role of uPAR in amoeboid angiogenesis in vivo. Before implantation we added to the plugs, composed of Matrigel plus physiologic protease-inhibitor cocktail, murine uPAR-aODN (ISIS Pharmaceuticals, Carlsbad Research Center, California) or M25 peptide used in vitro. At the third day, another dose of ODNs and M25 was injected subcutaneously into the plugs. Treatments consisted in plug administration of liposome-encapsulated vehicle alone (DOTAP; Roche, Germany), DOTAP + scramble ODN, DOTAP + uPAR-aODN, scramble M25 peptide and M25 peptide. Upon sacrifice (fifth day), isolated plugs were stained with hematoxylin-eosin.

### Statistical analysis

Unless otherwise stated, all the experiments were performed five times in duplicate for a reliable application of statistics. Statistical analysis was performed with GraphPad Prism5 software. Results are expressed as means ± SD. Multiple comparisons were performed by Anova and paired Student T test. Statistical significances were accepted at *p* < 0.05. (*p < 0,005, **p < 0,001, ***p < 0,0001).

## Results

### Induction of the amoeboid phenotype: Matrigel invasion, capillary morphogenesis, collagenolytic activity, cell morphology *and* Rac1/RhoA activation

**Fig. 1:**
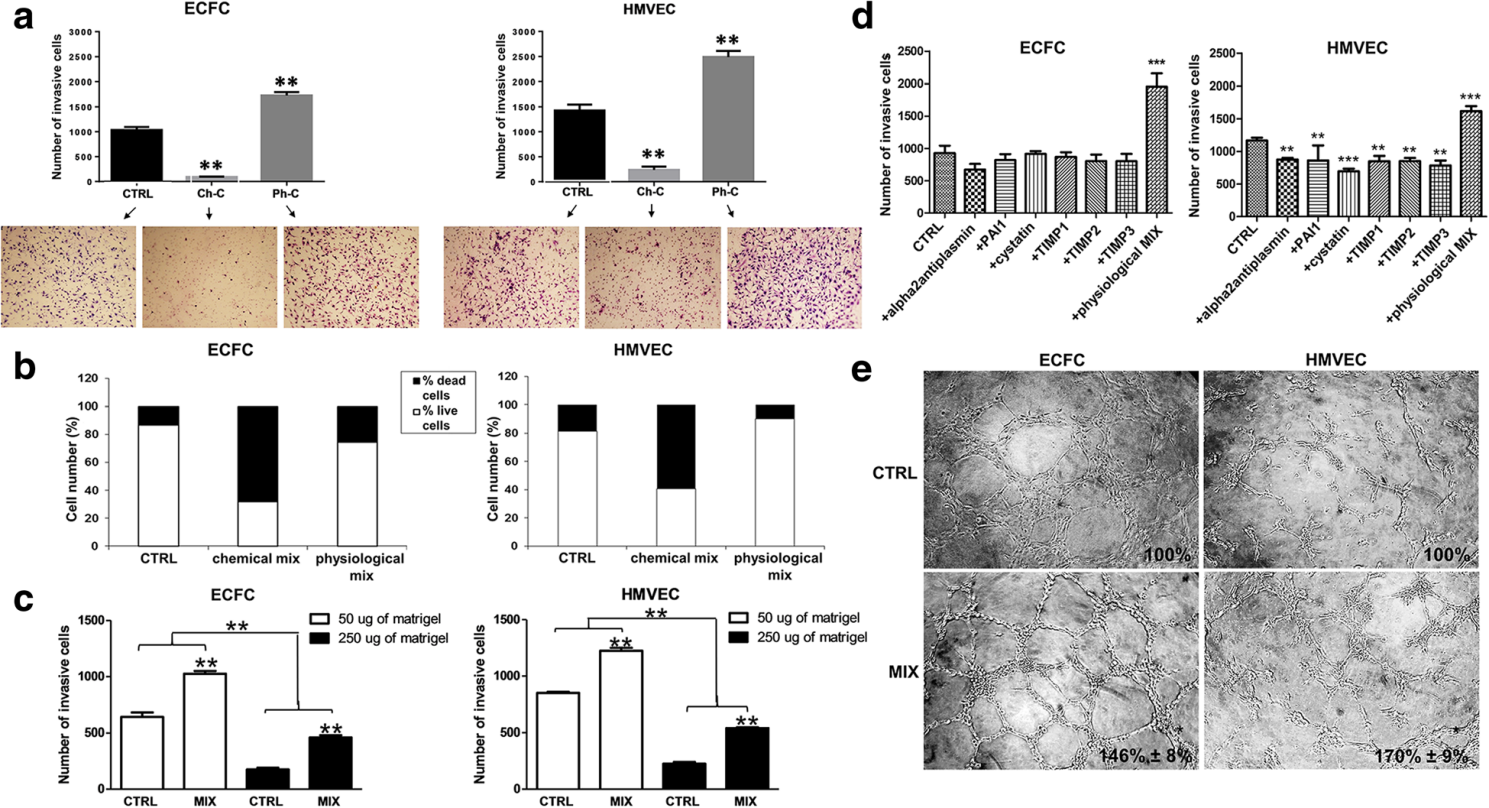
Induction of the amoeboid phenotype: Matrigel invasion and capillary morphogenesis. **a** Boyden chamber invasion assay through a thick Matrigel coating, in the presence of a chemical (Ch-C) or physiologic (Ph-C or MIX) protease inhibitor cocktail added to the Matrigel solution before polymerization. Histograms refer to quantification of Matrigel invasion assay obtained by counting the total number of migrated cells/filter. **b** ECFC and HMVEC cell viability upon protease inhibitor treatment after 6 and 24 (similar results not shown) hours evaluated by Trypan blue dye exclusion assay. The columns of histograms show in white the percentage of live cells and in black the percentage of dead cells. **c** ECFC and HMVEC invasion capacity in a Matrigel layer five times more concentrated (250 μg) than the usually used (50 μg). The ratio between the percentages of migrated cells in mesenchymal or amoeboid conditions after the increase of the Matrigel thickness doesn’t change. **d** Matrigel invasion assay in the presence of single inhibitors of the physiological MIX. Histograms refer to quantification of Matrigel invasion assay obtained by counting the total number of migrated cells/filter. **e** In vitro angiogenesis measured by capillary morphogenesis at 6 h in the presence and in the absence of the protease inhibitor MIX. Numbers on the lower right side of each picture indicate the percent field occupancy of capillary plexus as described in the Materials and Methods section. Quantification was performed at 6 h after seeding and was obtained by scanning of six to nine photographic fields for each condition. Results are the mean of 5 different experiments performed in duplicate, on two different clones derived from two different donors, on each cell line and are shown as mean value ± SD. *: *p* < 0.05; **: *p* < 0,001; ***p < 0,0001 significantly different from control

Selected families of membrane-associated proteases acting at specific steps of angiogenesis and vasculogenesis are required to perform a suitable angiogenic program [3, 20]. In order to investigate if the presence of protease inhibitors cocktail produced a protease-independent invasion in vitro, we first performed a Boyden chamber invasion assay. Notably, we added protease inhibitor cocktail to ECFC and HMVEC cell suspension and to Matrigel solution before polymerization. We used two different kinds of protease inhibitors cockatils: the chemical cocktail (Ch-C) [13, 21], and the physiological cocktail (Ph-C or MIX), composed as reported in M&M. Under the effect of the Ph-C both ECFCs and HMVECs showed a greatly enhanced Matrigel invasion, as opposed to the very poor movement and high toxicity observed with the Ch-C (Fig. 1a). Indeed, while the Ch-C proved to be very toxic for both ECFCs and HMVECs, the percent of cell death induced by the Ph-C was similar to that of untreated cells (Fig. 1b). Therefore, in the next experiments we always used the physiological cocktail that will be indicated as MIX. To be sure that the invasion capacity of endothelial cells in amoeboid conditions was independent from the compactness of the Matrigel, we tested the ECFC and HMVEC invasion capacity in a Matrigel layer five times more concentrated (250 μg) than the usually used (50 μg), observing that the ratio between the percentage of migrated cells in mesenchymal or amoeboid conditions was independent of the Matrigel density (Fig. 1c). Moreover, single inhibitors of the MIX produced no or scarce decrease of cell invasion as compared to the intense invasion-promoting activity of the full-range cocktail, demonstrating that the effect of the mix was due to the synergistic effect of all inhibitors mixed together and not to any biological activity of a single one at the used concentrations (Fig. 1d).

Capillary morphogenesis is considered a reliable in vitro analog of in vivo angiogenesis and has been recognized to be mainly dependent on MT1-MMP, MMP2, MMP9 and uPAR-bound uPA [22, 23]. We observed that both ECFCs and HMVECs produced similar tubular-like structures under mesenchymal or amoeboid conditions (Fig. 1e) and evaluated the speed of capillary structures formation by time-lapse capillary morphogenesis assay. It’s known that the low-adhesion attachment to the substrate enables cells that adopt an amoeboid movement to translocate at relatively high velocities [1]. We have observed that also the speed of tube formation for is higher in amoeboid conditions than in the presence of proteases (videos): at 25th second of the recorded video, corresponding to about 312 min, cells treated with the MIX (video: https://vimeo.com/246963233) formed a complex capillary network compared to the control (video: https://vimeo.com/246963182), with a ratio of about 2:1 evaluated counting the branching points formed.

**Fig. 2:**
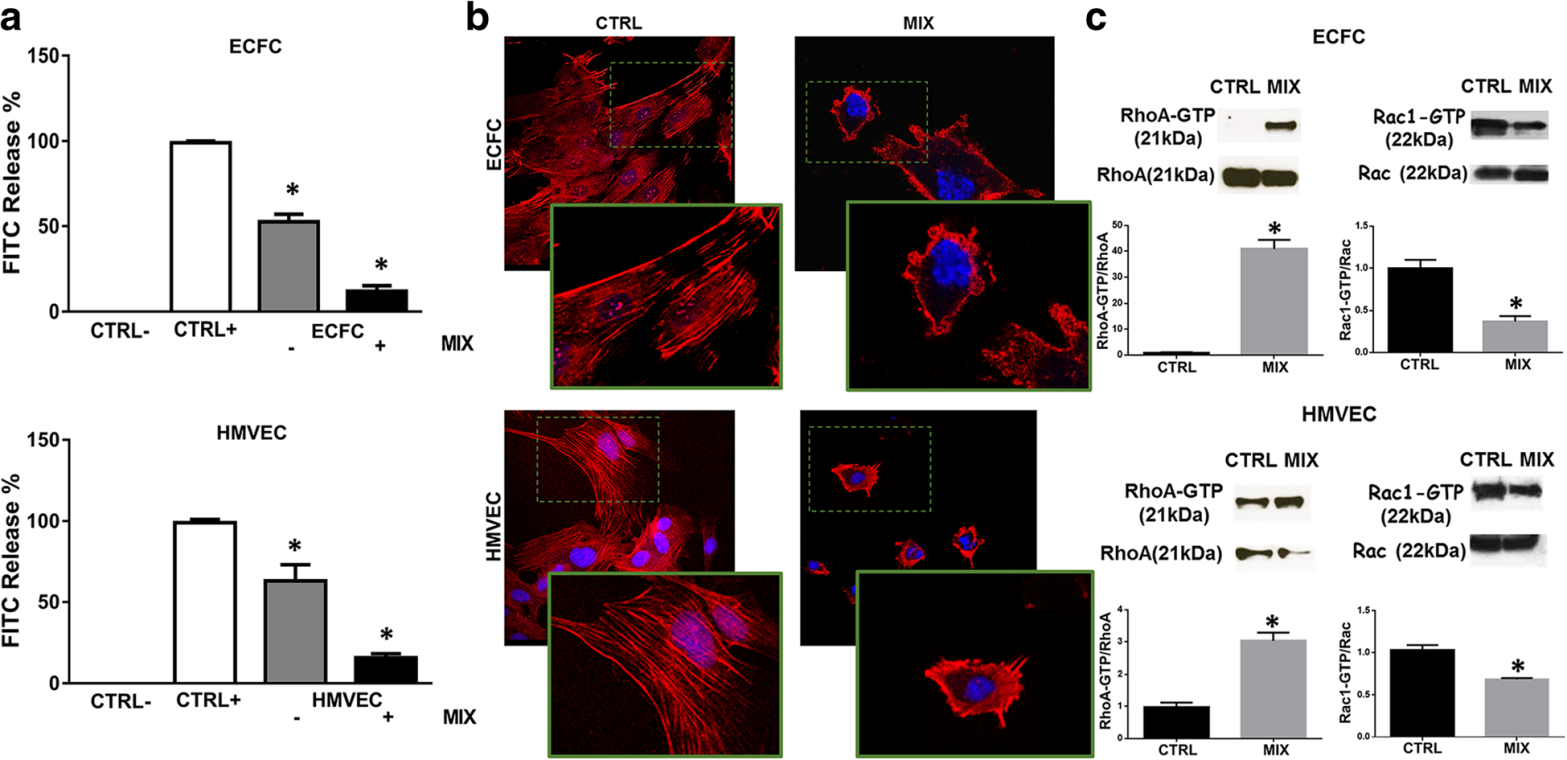
Induction of the amoeboid phenotype: cell morphology *and* Rac1/RhoA activation. **a** Histograms show the collagenolytic activity of ECFC and HMVEC cells under mesenchymal (-MIX) and amoeboid conditions (+MIX), expressed as % collagen degradation with respect to the positive control obtained by addition of exogenous collagenase. Ctrl-: collagenolytic activity in the absence of cells and exogenous collagen; Ctrl+: collagenolytic activity in the absence of cells but in the presence of exogenous collagenase; ECFC and HMVEC: collagenolytic activity in the presence of ECFCs or HMVECs. **b** Morphological features of the mesenchymal (elongated) to amoeboid (roundish) transition (MAT) of ECFCs and HMVECs. Each picture shows the general pattern and related magnification of a small field for each cell line. Red: phalloidin staining of the actin cytoskeleton. Blue: nuclear staining with DAPI. Magnification 40 X for reference pictures and 100 X for enlarged insets. Results shown are representative of two different preparations of each cell line under mesenchymal and amoeboid conditions. Sub-membranous cortical actin localization are evident chiefly in HMVECs and ECFCs. **c** Western blotting of total and GTP-loaded forms of small Rho-GTPasesRhoA and Rac1 under mesenchymal and amoeboid conditions for each cell line. RhoA-GTP and Rac1- GTP, GTP-loaded forms of small Rho GTP-ases; RhoA and Rac, total un-loaded forms of small Rho GTP-ases, used as a reference loading control. Numbers on the left refer to molecular weights expressed in kDa. Histograms report band densitometry. Results are the mean of 5 different experiments performed in duplicate, on two different clones derived from two different donors, on each cell line and are shown as mean value ± SD. *: *p* < 0.05 significantly different from control

As previously reported for melanoma and prostate cancer cells [13], the residual collagenolytic activity shown by endothelial cells under amoeboid conditions did not justify the number of invasive cells (Fig. 2a).

Cell morphology and its association with actin cytoskeleton assembly is a characteristic of the movement style. Mesenchymal motility is connoted by elongated, fibroblast-like cell morphology with established cell polarity, dependent on the small GTPase Rac which, in turn, organizes actin polymerization to form stress fibers, filopodia and lamellipodia [24], giving origin to actin-rich protrusions. These features were exhibited by both ECFCs and HMVECs under control mesenchymal conditions (Fig. 2b-c). Under protease inhibition, both cell lines acquired a round morphology with sub-membranous cortical actin localization, a feature connoting the amoeboid phenotype (Fig. 2c). Nevertheless, immune-phenotyping by FACS analysis (Table 1) revealed that both ECFCs and HMVECs, even after actin cytoskeletal reorganization, maintain endothelial characteristics.

**Table 1:** ECFC immunophenotyping by FACS analysis

Antigen	ECFC CTRL	ECFC MIX	HMVEC CTRL	HMVEC MIX
CD31	99,7 ± 0,3	99,8 ± 0,1	99,9 ± 0,1	100 ± 0,0
ULEX	98,9 ± 0,3	99,1 ± 0,4	99,5 ± 0,2	99,9 ± 0,1

Cell surface antigen expression. Results represent the mean percentage of cell expressing surface antigens ± SD from two different experiments performed on two different ECFC clones and two HMVEC lines

In order to feature the motility shift we also evaluated the activation of the small GTPases RhoA and Rac1, two accepted regulators of the cytoskeleton. It’s described that mesenchymal motility is associated with the inhibition of Rho GTPases and activation of Rac, which drives motility organizing actin polymerization and lamellipodium formation, whereas amoeboid motility is characterized by an opposite phenotype [25]. Western blotting and relative quantification for the activated forms of RhoA and Rac1, compared to the total amount of RhoA and Rac1, revealed an increase of activated RhoA and a decrease of activated Rac1, in both ECFCs and HMVECs after the induction of amoeboid motility in presence of the MIX (Fig. 2c).

### Amoeboid style of movement: role of uPAR

**Fig. 3:**
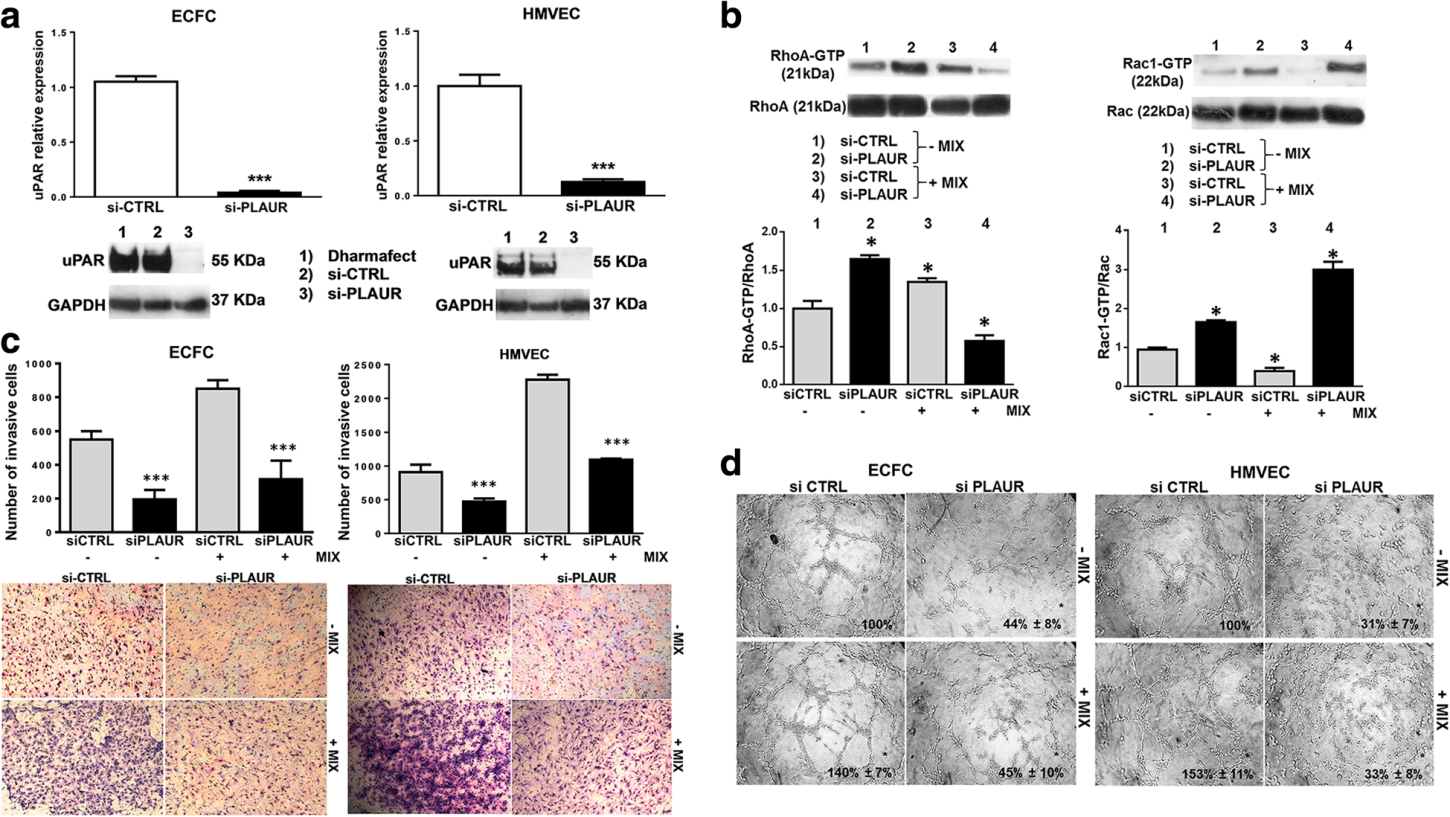
Effects of uPAR silencing on Rho-GTPases activation, invasion and capillary morphogenesis in mesenchymal, amoeboid conditions. **a** The upper panel shows quantitative Real-Time PCR of uPAR relative expression in both cell lines after siPLAUR treatment. Not-targeting siRNA pool constructs were used as negative control (siCONTROL). The lower panel shows western blotting analysis of uPAR for each cell line after siPLAUR treatment. Dharmafect: treatment of cells with the transfection reagent alone. Numbers on the left of each Western blotting refer to molecular weights expressed in kDa. **b** Western blotting of total and GTP-loaded forms of small Rho-GTPasesRhoA and Rac1 under mesenchymal and amoeboid conditions in ECFCs untreated and treated with siCTRL/siPLAUR, respectively. Histograms report RhoA-GTP/RhoA and Rac1-GTP/Rac1 ratio obtained by band densitometry quantification. The same experiment on HMVECs gave similar results (not shown). **c** Matrigel invasion under mesenchymal and amoeboid conditions untreated and treated with siCTRL/siPLAUR, respectively. Histograms refer to quantification of Matrigel invasion assay obtained by counting the total number of migrated cells/filter. **d** In vitro angiogenesis before and after uPAR silencing by siPLAUR was measured by capillary morphogenesis at 6 h in the presence and in the absence of the protease inhibitor MIX. Numbers on the lower right side of each picture indicate the percent field occupancy of capillary plexus as described in the Materials and Methods section. Quantification was performed at 6 h after seeding and was obtained by scanning of six to nine photographic fields for each condition. Results are the mean of 5 different experiments performed in duplicate, on two different clones derived from two different donors, on each cell line and are shown as mean value ± SD. *: *p* < 0.05; **: *p* < 0,001; ***p < 0,0001 significantly different from control

To investigate the role of uPAR in amoeboid angiogenesis, after validating the silencing activity of pooled small interfering RNAs targeting PLAUR mRNA (siPLAUR), that produced an evident reduction of uPAR expression in terms of mRNA and protein in both ECFCs and HMVECs (Fig. 3a), we have studied the effect of uPAR knockdown on the small Rho-GTPases activation. In mesenchymal conditions, siPLAUR treatment of ECFCs (Fig. 3b) and HMVECs (data not shown) resulted into increase of RhoA and Rac1 activation, whereas in the presence of inhibitor cocktail the same cells showed a reduced RhoA activation paralleled by an increased Rac1 activation. Therefore, even though after uPAR knockdown ECFCs and HMVECs still activate amoeboid/mesenchymal-related transductions, they are unable to invade 3D matrices (Fig. 3c) and to form tubular structures in vitro (Fig. 3d), indicating that both movement styles demand the presence of uPAR. The residual movement observed in the absence and in the presence of protease inhibitors, in cells treated with si-PLAUR, may possibly be ascribed to proteases and protease receptors of different families [26].

### uPAR-integrin interaction in amoeboid angiogenesis

**Fig. 4:**
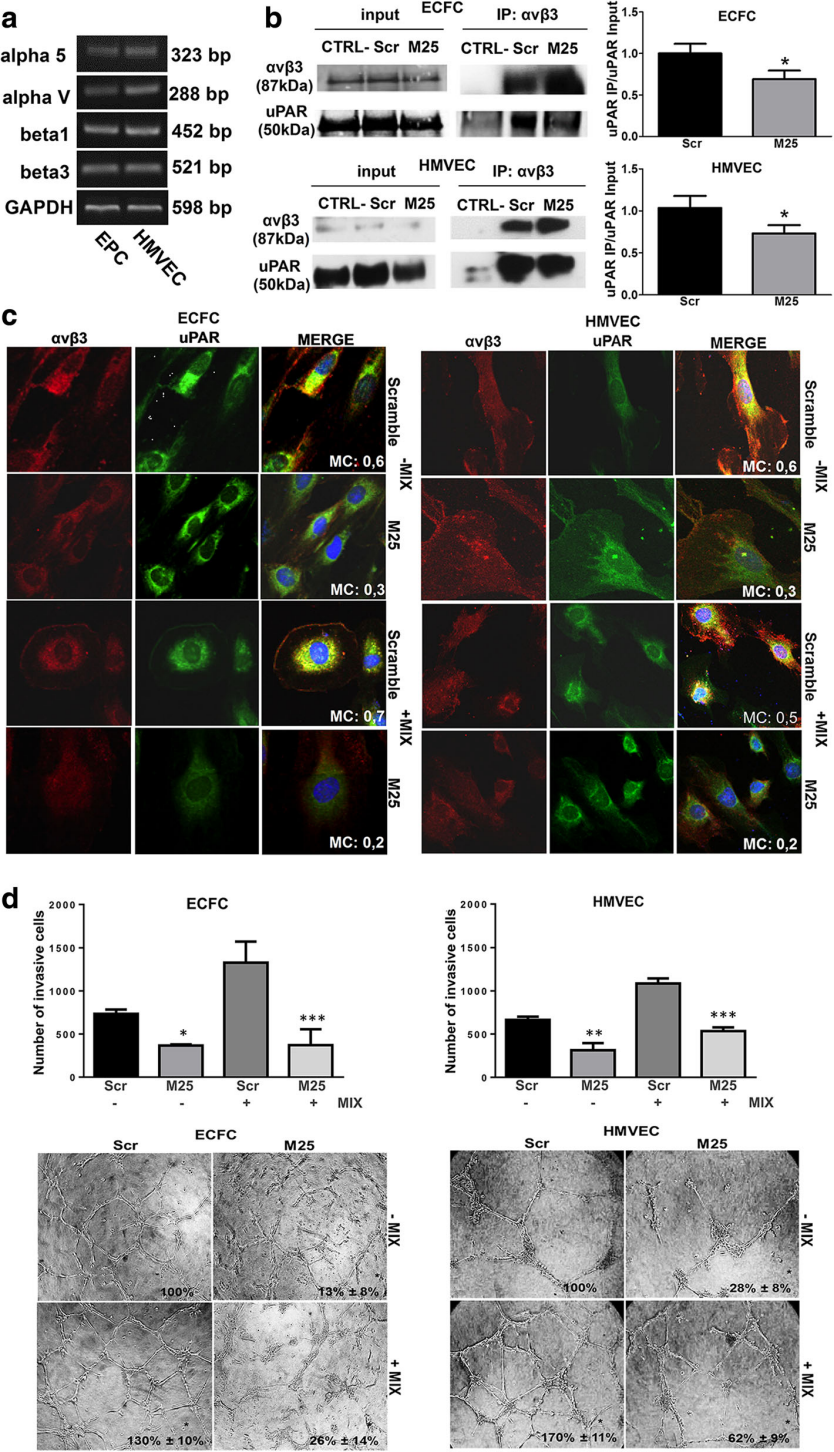
Integrin pattern and uPAR integrin interaction in amoeboid angiogenesis. **a** Semiquantitative PCR of the shown integrin α and β chains in ECFCs and HMVECs. GAPDH was used as a reference control. Product sizes, expressed in bp, are reported on the right. **b** Immunoprecipitation of αvβ3-integrin. Input: Western blotting of aliquots (30 μg of proteins) of cell lysates before immunoprecipitation, used as a reference loading control. IP αvβ3: immunoprecipitate (500 μg of proteins) obtained with anti-αvβ3-integrin antibody; alphav/beta3 lane: immunoblotting with anti-αvβ3 antibody; uPAR lane: immunoblotting with anti-uPAR antibody; CTRL-: a lysate that was treated with non-specific IgG (and Protein A/G) instead of the antibody and used as negative control. Molecular weights, expressed in kDa, are reported on the right. Histograms report band densitometry. Results are the mean of 3 different experiments performed in duplicate on each cell line and are shown as mean value ± SD. *: *p* < 0.05 significantly different from control **c** Confocal microscopy for αvβ3 integrin (red fluorescence) and uPAR (green fluorescence) co-localization in under mesenchymal (-MIX) and amoeboid (+MIX) conditions, in the absence and in the presence of M25 peptide and scramble M25 peptide (Scramble). Nuclear staining: DAPI (blue). The co-localization score is reported within each picture (MC: Manders’coefficient). Magnification: 40 X. The shown pictures are representative of 50 different pictures for each experimental condition and were studied by Image J analysis. **d** The upper panel shows Matrigel invasion under mesenchymal and amoeboid conditions untreated and treated with scramble M25 (Scr) and M25 peptide (M25), respectively. Histograms refer to quantification of Matrigel invasion assay obtained by counting the total number of migrated cells/filter. The lower panel shows in vitro angiogenesis at the same conditions described for Matrigel invasion. Numbers on the lower right side of each picture indicate the percent field occupancy of capillary plexus as described in the Materials and Methods section. Quantification was performed at 6 h after seeding and was obtained by scanning of six to nine photographic fields for each condition. Results are the mean of 5 different experiments performed in duplicate, on two different clones derived from two different donors, on each cell line and are shown as mean value ± SD. *: *p* < 0.05; **: *p* < 0,001; ***p < 0,0001 significantly different from control

ECFCs and HMVECs showed an integrin pattern in line with previous studies [18] (Fig. 4a). Immunoprecipitation experiments with lysates of ECFCs and HMVECs, demonstrated the activity of M25 peptide in uncoupling uPAR-integrin αvβ3 interaction (Fig. 4b). Figure 4c shows the confocal immune-fluorescence analysis of integrin αvβ3 and uPAR in both cell lines. These data showed that uPAR-integrin interactions persist under both mesenchymal and amoeboid conditions but, after cell treatment with 50 μM M25 peptide, we assist to an uncoupling between uPAR and αvβ3 integrin in the absence and in the presence of the inhibitor- IX, resulting in abolition of mesenchymal and amoeboid invasion and angiogenesis as shown by invasion assay and capillary morphogenesis (Fig. 4d), Taken together, these results indicate a role of the uPAR-integrin-actin axis in the regulation of amoeboid angiogenesis.

### VEGF role in amoeboid angiogenesis

Endothelial cells respond to the most potent of the proangiogenic regulators, the vascular endothelial growth factor (VEGF), that binds VEGF receptors on the cell surface stimulating endothelial cell migration and proliferation, new blood vessels formation and sprouting, and triggers the caveolar-raft recruitment of proteins able to maintain a proper angiogenic function of endothelial cells and preserve the integrity of the actin cytoskeleton [27].

Here we examined the role in amoeboid conditions of VEGF and VEGFR2/KDR/Flk-1, considered the major mediator of proangiogenic signaling. In endothelial cells VEGF controls the delivery of KDR from the endosomal storage pool to the plasma membrane [28].

**Fig. 5:**
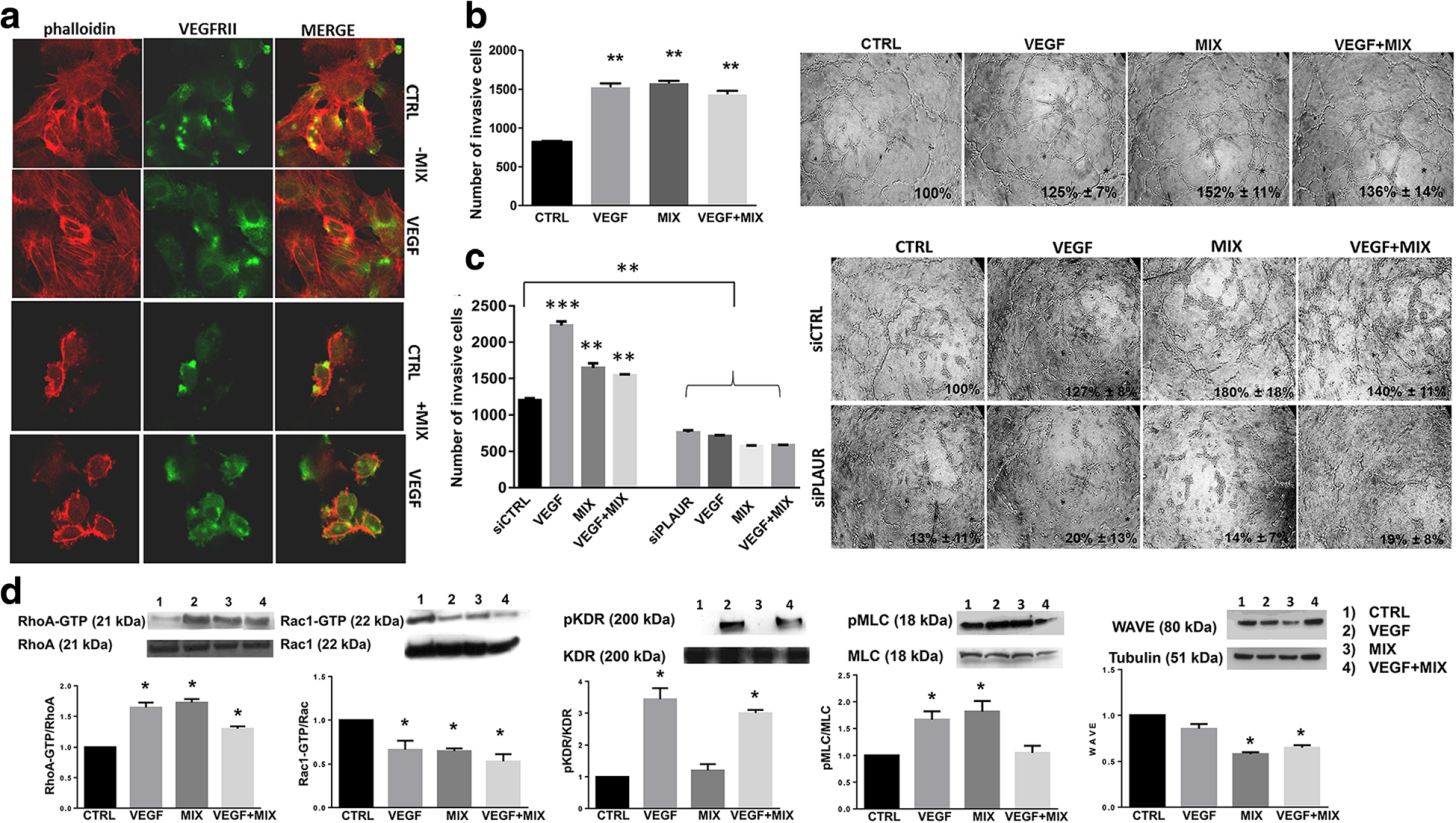
VEGF role in amoeboid angiogenesis. **a** Confocal microscopy for F-actin by phalloidin staining (red fluorescence) and VEGFRII (green fluorescence) under mesenchymal (-MIX) and amoeboid (+MIX) conditions, in the absence and in the presence of VEGF. Magnification: 40 X. Phalloidin was used to make more evident the cell membrane profile under amoeboid conditions. **b** Histogram on the left refers to quantification of Matrigel invasion assay obtained by counting the total number of migrated cells/filter. The assay was performed in the presence and in the absence of the MIX added to the Matrigel solution before polymerization and after addition of VEGF in the cell suspension. On the right capillary morphogenesis performed at the same conditions described for Matrigel invasion assay. Numbers on the lower right side of each picture indicate the percent field occupancy of capillary plexus as described in the Materials and Methods section. Quantification was performed at 6 h after seeding and was obtained by scanning of six to nine photographic fields for each condition. **c** Hystogram on the left shows results from boyden chamber invasion assay through a thick Matrigel coating in mesenchymal and amoeboid conditions, before and after uPAR silencing and with and without VEGF stimulation. siCTRL: negative control. siPLAUR: specific siRNA smart pools directed to uPAR gene. On the right capillary morphogenesis performed at the same conditions described for Matrigel invasion assay. **d** Western blotting results show the effects of VEGF, in mesenchymal and amoeboid conditions, on the intracellular signaling molecules RhoA and Rac1, the phosphorylation of KDR and MLC2 and WAVE2. Numbers on the left refer to molecular weights expressed in kDa. Histograms report band densitometry. Results are the mean of 5 different experiments performed in duplicate, on two different clones derived from two different donors, on each cell line and are shown as mean value ± SD. *: *p* < 0.05; **: *p* < 0,001; ***p < 0,0001 significantly different from control. Figure 5 shows results obtained with ECFCs. HMVECs gave similar results (not shown)

We first examined the cellular distribution of endogenous KDR in ECFCs in mesenchymal conditions before and after VEGF stimulation. Under control conditions, KDR showed mainly a clustered staining pattern in the cytoplasm. VEGF stimulation leaded to a redistribution of KDR, which lost the clustered pattern and spread within the cytoplasm to reach the plasma membrane, with no substantial differences between mesenchymal and amoeboid conditions (Fig. 5a).

Figure 5b-c show an “indifference” of ECFCs to VEGF stimulation under amoeboid conditions in terms of invasion and capillary morphogenesis. While Matrigel invasion under mesenchymal conditions was dependent on the chemotactic activity of VEGF, amoeboid invasion was VEGF-indifferent (Fig. 5b). Similar results were obtained in capillary morphogenesis assay (Fig. 5b), possibly due to the endothelial cells capacity to migrate and differentiate into tubular structures at the maximum levels under amoeboid conditions. We performed the same experiments of migration and capillary morphogenesis after uPAR silencing. uPAR knockdown greatly impaired ECFC invasion and tubular structure formation in the presence of VEGF with or without MIX in vitro, indicating that both movement styles, also under VEGF stimulation, demand the presence of uPAR (Fig. 5c).

We then investigated the effect of VEGF on the intracellular signaling molecules like the small GTPases RhoA and Rac1, pKDR, pMLC2 and WAVE2 (Fig. 5d). Our results reveal that VEGF seems to induce an amoeboid signaling pattern in both mesenchymal and amoeboid conditions, as we can see from the results of Rho and Rac activity assay. We found obviously an increment of pKDR after VEGF stimulation in both conditions. As we anticipated above, in the presence of inhibitor-MIX, even though VEGF signaling is active, the cells can’t use that stimulus because they already invade and differentiate to the maximum levels. Because amoeboid movement is associated with elevated levels of Myosin Light Chain 2 (MLC2) phosphorylation, we found an increment in the presence of protease inhibitor-MIX and, under VEGF stimulation, elevated levels in mesenchymal conditions compared to the control and a reduction in amoeboid conditions. WAVE2 is responsible for downregulation of amoeboid motility and therefore of actomyosin contractility and membrane blebbing. We indeed found a downregulation in the presence of inhibitor MIX, before and after VEGF treatment, compared to the control in mesenchymal conditions. After VEGF treatment in mesenchymal conditions, WAVE2 expression is similar to the control. Each result shown in Fig. 5 reveals representative data obtained with ECFCs. HMVECs gave similar results (not shown).

### In vivo evidence of amoeboid angiogenesis in the Matrigel plug assay

**Fig. 6:**
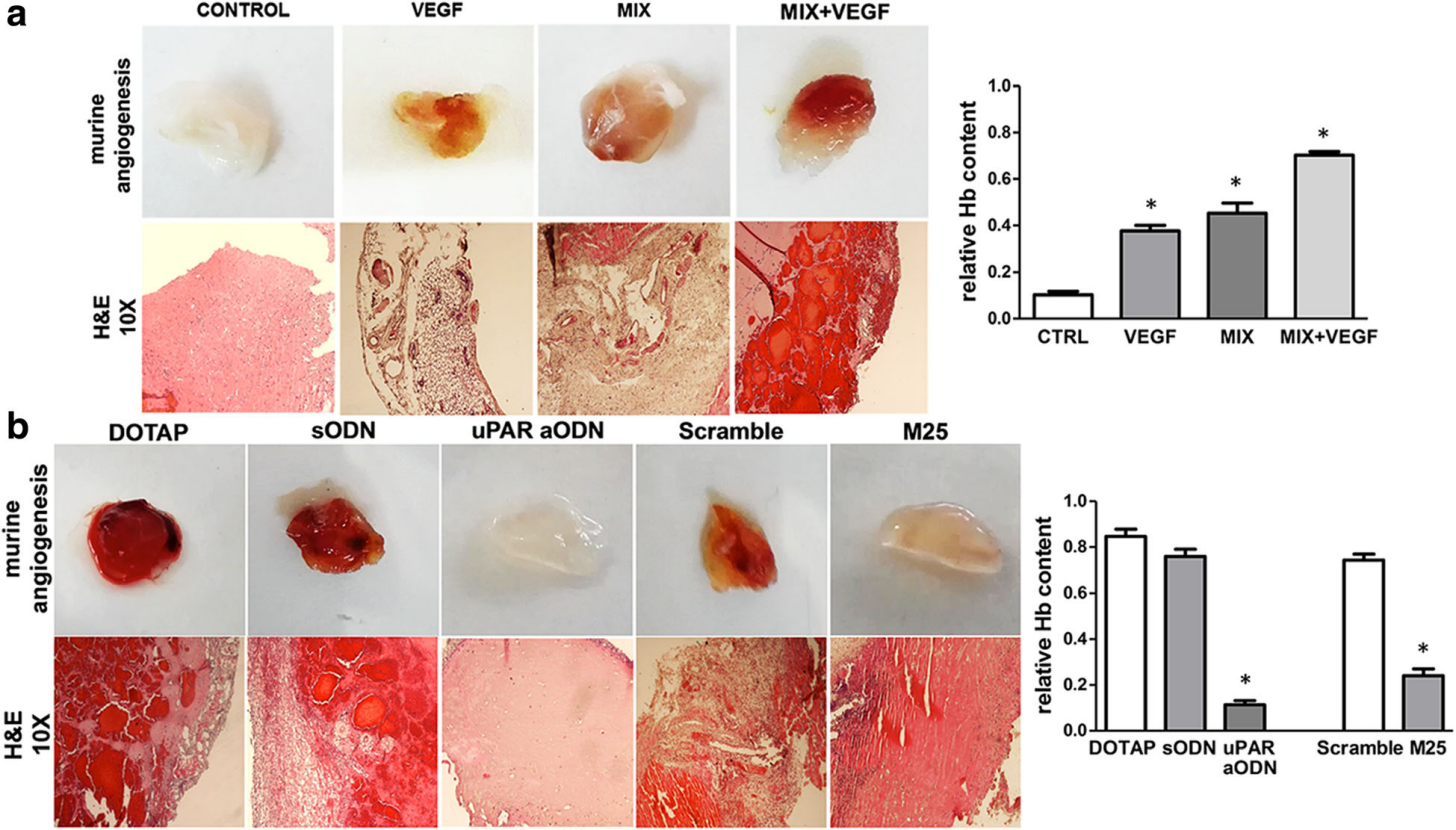
In vivo amoeboid angiogenesis. Effects of uPAR silencing and uPAR-integrin uncoupling in Matrigel Plug Assay. Angiogenesis in a Matrigel plug assay in SCID mice by the subcutaneosly addition of Matrigel containing heparin (50 U/ml) with and without VEGF in both mesenchymal and amoeboid conditions. **a** In the first lane, a representative photograph of individual Matrigel plugs recovered at autopsy for each condition shown. Angiogenesis was evaluated by hemoglobin (Hb) contents shown in the histograms on the right. Consecutive 5 μm histological sections from Matrigel plugs were recovered 5 days after implantation. The second lane shows hematoxylin/eosin staining. **b** Angiogenesis in a Matrigel plug assay in SCID mice by the subcutaneosly addition of Matrigel containing heparin (50 U/ml) and protease inhibitor mix. Treatments consisted in plug administration of liposome-encapsulated vehicle alone (DOTAP), DOTAP + scramble ODN (sODN), DOTAP + uPAR-aODN (uPARaODN), scramble M25 peptide (Scramble) and M25 peptide (M25). In the first lane, a representative photograph of individual Matrigel plugs recovered at autopsy for each condition shown. Angiogenesis was evaluated by hemoglobin (Hb) contents shown in the histograms on the right. Consecutive 5 μm histological sections from Matrigel plugs were recovered 5 days after implantation. The second and the third lane show hematoxylin/eosin. Images were captured at Å~ 10 magnification. Pictures shown in the figures (**a**) and (**b**) are representative of 2 different experiments performed in duplicate on two mice for each experimental condition, each mouse injected in both flanks

We performed the Matrigel plug assay in SCID mice to study a possible role of the amoeboid movement in vivo. In the first in vivo experiment, we used 8 SCID mice to examine murine angiogenesis in control conditions and after addition of inhibitor-MIX to unpolymerized Matrigel, both in the presence and in the absence of VEGF. Individual Matrigel plugs were recovered at autopsy 5 days after implants. Vascularization was evaluated by sight taking a representative photograph, of individual Matrigel plugs recovered at autopsy for the corresponding condition and after histological analysis (Fig. 6).

The plugs recovered from mice showed that whereas control conditions displayed trace of vessels, the inhibitor-MIX stimulated angiogenesis, as shown also by increases in hemoglobin content. This effect was the result of protease inhibitor activity and was particularly relevant when VEGF was added to the MIX (Fig. 6a). Hematoxylin/eosin staining revealed that plugs containing the protease inhibitor MIX showed a strong angiogenic response with an increased number of vascular structures with lumens and red blood cells as compared to control plugs.

In the second experiment we performed another Matrigel plug assay using 10 SCID mice to show uPAR dependence in amoeboid angiogenesis in vivo. We evaluated uPAR silencing by murine uPAR-aODN and uPAR-integrin uncoupling by the M25 peptide, already reported to efficiently inhibit mesenchymal angiogenesis [13] and invasion of tumor cells [14]. We added both the relevant molecules (uPAR-aODN and M25 peptide) and the respective negative controls to unpolymerized Matrigel, containing inhibitor-MIX, before implantation. At the third day, other treatments were performed subcutaneously into the plugs: such treatments consisted in plug administration of liposome-encapsulated vehicle alone (DOTAP), DOTAP + scramble ODN, DOTAP + uPAR-aODN, scramble M25 peptide and M25 peptide. As shown in Fig. 6b, uPAR-integrin uncoupling by the M25 peptide as well as uPAR silencing produced an evident reduction of amoeboid angiogenesis, as shown also by decreases in hemoglobin content, indicating a role of uPAR and uPAR-integrin-actin axis in the regulation of amoeboid angiogenesis also in vivo. These results were confirmed in histological analysis after staining with hematoxylin-eosin that revealed the presence of murine vessels.

## Discussion

We have shown that ECs and ECFCs enter into a so far undescribed program of vessel formation, that we call amoeboid angiogenesis, when protease inhibitors overwhelm their proteolytic potential.

An interesting paper by Stratman et al. [22] reported that in 3D matrices ECs exploit MT1-MMP proteolytic activity to excavate vascular guidance tunnels during tube morphogenesis. Afterwards, ECs migrate within such tunnels even upon inhibition of MMP activity. However, a possible amoeboid movement of ECFCs has been suggested only on the basis of cell morphology [1, 11, 29, 30]. A recent paper [31] has described an amoeboid phase of endothelial cells engaged in the endothelial to mesenchymal transition in the frame of Systemic Sclerosis-associated fibrosis.

In a previous study we have shown that, in malignant melanoma and prostate cancer, the receptor of the urokinase-plasminogen-activator (uPAR, CD87) is indispensable for supporting a shift from a mesenchymal to an amoeboid movement [13]: uPAR regulates cortical actin contraction/relaxation cycles by interaction with beta1 and beta3 integrins, that in turn connect uPAR to the actin in a RGD-independent fashion. This happens even in the presence of a full-range of synthetic proteases-inhibitor cocktail.

Here we show that both mature ECs and ECFCs can migrate by mesenchymal and amoeboid style. For this purpose, we used a mixture of physiologic inhibitors of serine-proteases, metallo-proteases and cysteine-proteases, thus mimicking a possible physiological environment that ECs and ECFCs may encounter during their migration within tissues. This study shows that, under the effect of the physiological inhibitor-MIX, both ECFCs and HMVECs performed a greatly enhanced Matrigel invasion, producing tube-like structure as in mesenchymal conditions. The protease inhibitor-MIX used in this study is composed by molecules that individually affect a broad range of substrates, including molecules other than proteases [12, 32–35]. Here we demonstrated that the biological activity of the single inhibitors produced no or scarce decrease of cell invasion as compared to the intense invasion-promoting activity of the full-range cocktail. This is possibly related to the described cross-talks among the main families of proteases, whereby both serine-proteases and cathepsins may activate pro-uPA to uPA [36], and plasmin is involved in proteolytic activation of pro-MMP1 [37], pro-MMP3 [38], pro-MMP9 [39]. Therefore, we additionally demonstrated that the effect of the mix was due to the synergistic effect of all inhibitors mixed together and not to any biological activity of a single one. Moreover, a large body of literature indicates that each inhibitor exhibited protease inhibition-independent activities that often resulted into angiogenesis inhibition, and never in its stimulation. These observations indicate that the endothelial cell amoeboid invasion and angiogenesis are an environment-dependent escape mechanism.

We also had in vivo evidence of amoeboid angiogenesis in the Matrigel plug Assay confirming that the inhibitor-MIX stimulated the in vivo angiogenesis showing an increased number of vascular structures with lumens and red blood cells. We have been unable to avoid a hemorrhagic effect under amoeboid conditions in the presence of VEGF. We speculated this is related to the VEGF effect as vascular permeability factor acting on the tumultuous and fast angiogenesis dictated by amoeboid conditions.

These findings indicate the existence of a new type of neovascularization: the “amoeboid angiogenesis”. uPAR silencing with aODN and uPAR-integrin uncoupling with the M25 peptide abolished both mesenchymal and amoeboid invasion and angiogenesis of ECs and ECFCs in vitro and amoeboid murine angiogenesis in vivo, indicating a role of the uPAR-integrin-actin axis in the regulation of amoeboid angiogenesis.

However, since uPAR silencing either in melanoma and prostate cancer cells [13] or in ECs and ECFCs is unable to exhaustively inhibit amoeboid movement, we speculate on a possible role of other protease receptors (in particular MMPs receptors), in the light of the recent observation indicating that also MMP9 regulates amoeboid migration of melanoma cells in a catalytic independent manner through regulation of actomyosin contractility via its CD44 receptor [26].

Confocal immuno-fluorescence analysis of integrin αvβ3 and uPAR showed that uPAR-integrins interactions persist under both mesenchymal and amoeboid conditions. Treatment of cells with 50 μM peptide M25 uncoupled uPAR from integrins, in the absence and in the presence of the inhibitor cocktail demonstrating that in ECFCs and ECs, uPAR signaling is mediated by αvβ3 integrins also in amoeboid conditions.

The targeting of vascular endothelial growth factor A (VEGFA), a crucial regulator of both normal and pathological angiogenesis, resulted in innovative therapeutic approaches in oncology and ophthalmology [40, 41] and, in combination with chemotherapy and radiation, is able to correct leaking vessels, to decrease tumor interstitial pressure, and to inhibit vessels development. Although VEGF-targeted therapies currently are standard of therapy for multiple tumor types, many patients develop resistance and progress toward metastasis. In this context, targeting both VEGF in parallel with other pathways implicated in angiogenesis should result into more effective tumor growth inhibition. Here we tested VEGF stimulation under amoeboid conditions and observed an “indifference” of ECs and ECFCs to VEGF treatment. Maybe it could be ascribed to the endothelial cells capacity to migrate and differentiate into tubular structures at the maximum levels under amoeboid conditions thus justifying the limited efficacy of VEGF-targeted therapies as detailed above. Furthermore, surprisingly, our results reveal that VEGF stimulation induce an amoeboid-like signaling pattern in both mesenchymal and amoeboid conditions.

Synthetic metalloproteinase inhibitors (MPIs) were developed and utilized in human clinical trials but the results were disappointing [42]. MPIs are now viewed only as potential antiangiogenic agents for primary tumors [43] and as a therapy able to maintain small clusters of metastatic cells in a dormant state. In light of what we have shown up in this study, we hypothesized that the failure of treatment after the initial stages of tumor development could be ascribed also to the onset of the angiogenic transition, during which the tumor microenvironment is able to skip the attack of the MPI therapy by allowing blood vessel formation using the “amoeboid” strategy.

So far, there are no additional explanations about the mechanisms that determine the transition from one migration mode to another, excluding the presence or absence of protease inhibitors. It is known that the so-called cell “protease web” (both membrane-associated and released into the ECM), reaches a high level of complexity, involving the whole range of proteases and about 140 substrates, many of which deal with angiogenesis [44]. In particular, MMPs may release from ECM many biologically active protein fragments and the main growth factors involved in angiogenesis, VEGF, FGF2, TGF-β, that pave the way to angiogenic endothelial cells, also providing a chemotactic gradient for the growing vessel. All such factors induce actin stress fibers organization and the release of pro-angiogenic proteases, features that define a “mesenchymal” mode of endothelial cell invasion [45–47]. The presence of a full-range protease inhibitor cocktail blocks availability of ECM-trapped angiogenic molecules, an event that triggers the amoeboid migration, an ancestral escape mechanism of movement ranging from amoebae to vertebrates [48].

Supporting our thesis, recent studies demonstrate that TIMP family members (TIMP1, TIMP2, TIMP3), as well as other physiological protease inhibitors (PAI-1, alpha2-antiplasmin, cystatin), are regarded as negative prognostic factors in patients and in experimental animals showing increased plasma and intra-tumor concentrations [49–55]. Even the assumption that the natural protease inhibitors could exhibit an anti-metastatic effect had been challenged in the past few years. For example, despite its function as an inhibitor of urokinase and tissue-type plasminogen activator (PA), PA inhibitor-1 (PAI-1) has a paradoxical pro-tumorigenic role in cancer, promoting angiogenesis and tumor cell survival and, as a biomarker in breast cancer, is validated for prognostic use in level-of-evidence-1 studies [35]. Our results, along with the results obtained in other studies [56, 57], show that uPAR is a molecular mediator of plasticity in angiogenesis, in concert with integrins [58]. Our results, along with the results obtained in other studies [56, 57], show that uPAR is an indispensable molecular mediator of plasticity in angiogenesis, in concert with integrins [58]. Besides, Rao and coworkers [59] demonstrated that a cooperation between uPA/uPAR and MMP-9 is required for breaching of the vascular wall, a rate-limiting step for intravasation, and consequently for tumor progression and metastasis. Therefore, uPAR silencing or the block of its interaction with integrins, together with standard treatment against VEGF, could be a possible therapeutic strategy impairing vascular growth and cancer cell invasion at the same time, overcoming resistance to anti-VEGF and anti-protease therapy.

## Conclusions

Taken together, our data show that in a microenvironment enriched with full-range protease inhibitors (for metallo- serine- and cysteine-proteinases) ECs acquire a round shape, connoted by sub-cortical actin localization and RhoA activation. Moreover, we demonstrate that the amoeboid movement of ECs depends on uPAR/integrin αvβ3 interaction and may be controlled by a 25mer peptide that inhibits uPAR/integrin contacts. Lastly, we have called this new adaptive program “amoeboid angiogenesis” and have ascertained that it is VEGF-independent and twice faster than the protease-dependent mesenchymal one.

## Additional file

Additional file 1: Supplementary Materials and Methods. (DOCX 18 kb)

## References

[1] Friedl P (2004). Prespecification and plasticity: shifting mechanisms of cell migration. Curr Opin Cell Biol.

[2] Adams RH, Alitalo K (2007). Molecular regulation of angiogenesis and lymphangiogenesis. Nat Rev Mol Cell Biol.

[3] Laurenzana A, Fibbi G, Chillà A (2015). Lipid rafts: integrated platforms for vascular organization offering therapeutic opportunities. Cell Mol Life Sci.

[4] Nobes CD, Hall A (1995). Rho, rac, and cdc42 GTPases regulate the assembly of multimolecular focal complexes associated with actin stress fibers, lamellipodia, and filopodia. Cell.

[5] Wolfenson H, Henis YI, Geiger B (2009). The heel and toe of the cell's foot: a multifaceted approach for understanding the structure and dynamics of focal adhesions. Cell Motil Cytoskeleton.

[6] vanHinsbergh VW, Engelse MA, Quax PH (2006). Pericellular proteases in angiogenesis and vasculogenesis. Arterioscler Thromb Vasc Biol.

[7] Friedl P, Borgmann S, Bröcker EB (2001). Amoeboid leukocyte crawling through extracellular matrix: lessons from the Dictyostelium paradigm of cell movement. J Leukoc Biol.

[8] Wang WY, Chien YC, Jan JS (2002). Consistent sequence variation of Epstein-Barr virus nuclear antigen 1 in primary tumor and peripheral blood cells of patients with nasopharyngeal carcinoma. Clin Cancer Res.

[9] Wyckoff JB, Pinner SE, Gschmeissner S (2006). ROCK- and myosin-dependent matrix deformation enables protease-independent tumor-cell invasion in vivo. Curr Biol.

[10] Sanz-Moreno V, Gadea G, Ahn J (2008). Rac activation and inactivation control plasticity of tumor cell movement. Cell.

[11] Pelosi E, Valtieri M, Coppola S (2002). Identification of the hemangioblast in postnatal life. Blood.

[12] Laurenzana A, Fibbi G, Margheri F (2015). Endothelial progenitor cells in sprouting angiogenesis: proteases pave the way. Curr Mol Med.

[13] Margheri F, Luciani C, Taddei ML (2014). The receptor for urokinase-plasminogen activator (uPAR) controls plasticity of cancer cell movement in mesenchymal and amoeboid migration style. Oncotarget.

[14] Margheri F, Chillà A, Laurenzana A (2011). Endothelial progenitor cell-dependent angiogenesis requires localization of the full-length form of uPAR in caveolae. Blood.

[15] Margheri F, Papucci L, Schiavone N (2015). Differential uPAR recruitment in caveolar-lipid rafts by GM1 and GM3 gangliosides regulates endothelial progenitor cells angiogenesis. J Cell Mol Med.

[16] Brodsky SV, Malinowski K, Golightly M, Jesty J, Goligorsky MS (2002). Plasminogen activator inhibitor-1 promotes formation of endothelial microparticles with procoagulant potential. Circulation.

[17] Ikenaka Y, Yoshiji H, Kuriyama S (2003). Tissue inhibitor of metalloproteinases-1 (TIMP-1) inhibits tumor growth and angiogenesis in the TIMP-1 transgenic mouse model. Int J Cancer.

[18] Margheri F, Manetti M, Serrati S (2006). Domain 1 of the urokinase-type plasminogen activator receptor is required for its morphologic and functional, beta2 integrin-mediated connection with actin cytoskeleton in human microvascular endothelial cells: failure of association in systemic sclerosis endothelial cells. Arthritis Rheum.

[19] Simon DI, Wei Y, Zhang L (2000). Identification of a urokinase receptor-integrin interaction site. Promiscuous regulator of integrin function. J Biol Chem.

[20] Verloop RE, Koolwijk P, van Zonneveld AJ (2009). Proteases and receptors in the recruitment of endothelial progenitor cells in neovascularization. Eur Cytokine Netw.

[21] Parri M, Taddei ML, Bianchini F (2009). EphA2 reexpression prompts invasion of melanoma cells shifting from mesenchymal to amoeboid-like motility style. Cancer Res.

[22] Stratman AN, Saunders WB, Sacharidou A (2009). Endothelial cell lumen and vascular guidance tunnel formation requires MT1-MMP-dependent proteolysis in 3-dimensional collagen matrices. Blood.

[23] Senger DR, Davis GE (2011). Angiogenesis. Cold Spring Harb Perspect Biol.

[24] Ridley AJ, Schwartz MA, Burridge K (2003). Cell migration: integrating signals from front to back. Science.

[25] Yamazaki D, Kurisu S, Takenawa T (2005). Regulation of cancer cell motility through actin reorganization. Cancer Sci.

[26] Orgaz JL, Pandya P, Dalmeida R (2014). Diverse matrix metalloproteinase functions regulate cancer amoeboid migration. Nat Commun.

[27] Chillà A, Magherini F, Margheri F (2013). Proteomic identification of VEGF dependent protein enrichment to membrane caveolar-raft microdomains in endothelial progenitor cells. Mol Cell Proteomics.

[28] Gampel A, Moss L, Jones MC (2006). VEGF regulates the mobilization of VEGFR2/KDR from an intracellular endothelial storage compartment. Blood.

[29] Quirici N, Soligo D, Caneva L. et al. Differentiation and expansion of endothelial cells from human bone marrow CD133(+) cells. Br J Haematol. 2001;115(1):186–94. 10.1046/j.1365-2141.2001.03077.x 11722432

[30] Guo H, Fang B, Liao L (2003). Hemangioblastic characteristics of fetal bone marrow-derived Flk1(+)CD31(−)CD34(−) cells. Exp Hematol.

[31] Kryczka J, Przygodzka P, Bogusz H (2017). HMEC-1 adopt the mixed amoeboid-mesenchymal migration type during EndMT. Eur J Cell Biol.

[32] Stetler-Stevenson WG (2008). Tissue inhibitors of metalloproteinases in cell signaling: metalloproteinase-independent biological activities. Sci Signal.

[33] Seo DW, Li H, Guedez L (2003). TIMP-2 mediated inhibition of angiogenesis: an MMP-independent mechanism. Cell.

[34] Jackson HW, Defamie V, Waterhouse P (2017). TIMPs: versatile extracellular regulators in cancer. Nat Rev Cancer.

[35] Duffy MJ, McGowan PM, Harbeck N (2014). uPA and PAI-1 as biomarkers in breast cancer: validated for clinical use in level-of-evidence-1 studies. Breast Cancer Res.

[36] Goretzki L, Schmitt M, Mann K, Calvete J, Chucholowski N, Kramer M (1992). Effective activation of the proenzyme form of the urokinase-type plasminogen activator (pro-uPA) by the cysteine protease cathepsin L. FEBS Lett.

[37] Murphy G, Stanton H, Cowell S (1999). Mechanisms for pro matrix metalloproteinase activation. APMIS.

[38] Ramos-DeSimone N, Hahn-Dantona E, Sipley J, Nagase H, French DL, Quigley JP (1999). Activation of matrix metalloproteinase-9 (MMP-9) via a converging plasmin/stromelysin-1 cascade enhances tumor cell invasion. J Biol Chem.

[39] Legrand C, Polette M, Tournie JM (2001). uPA/plasmin system-mediated MMP-9 activation is implicated in bronchial epithelial cell migration. Exp Cell Res.

[40] Ferrara N, Adamis AP (2016). Ten years of anti-vascular endothelial growth factor therapy. Nat Rev Drug Discov.

[41] Lulli M, Cammalleri M, Fornaciari I, Casini G, Dal Monte M (2015). Acetyl-11-keto-β-boswellic acid reduces retinal angiogenesis in a mouse model of oxygen-induced retinopathy. Exp Eye Res.

[42] Coussens LM, Fingleton B, Matrisian LM (2002). Matrix metalloproteinase inhibitors and cancer: trials and tribulations. Science.

[43] Stetler-Stevenson WG (1999). Matrix metalloproteinases in angiogenesis: a moving target for therapeutic intervention. J Clin Invest.

[44] Rodríguez D, Morrison CJ, Overall CM (2010). Matrix metalloproteinases: what do they not do? New substrates and biological roles identified by murine models and proteomics. Biochim Biophys Acta.

[45] Rousseau S, Houle F, Kotanides H (2000). Vascular endothelial growth factor (VEGF)-driven actin-based motility is mediated by VEGFR2 and requires concerted activation of stress-activated protein kinase 2 (SAPK2/p38) and geldanamycin-sensitive phosphorylation of focal adhesion kinase. J Biol Chem.

[46] Lamalice L, Le Boeuf F, Huot J (2007). Endothelial cell migration during angiogenesis. Circ Res.

[47] Curado F, Spuul P, Egaña I (2014). ALK5 and ALK1 play antagonistic roles in transforming growth factor β-induced podosome formation in aortic endothelial cells. Mol Cell Biol.

[48] Liu YJ, Le Berre M, Lautenschlaeger F (2015). Confinement and low adhesion induce fast amoeboid migration of slow mesenchymal cells. Cell.

[49] Vizoso FJ, González LO, Corte MD (2007). Study of matrix metalloproteinases and their inhibitors in breast cancer. Br J Cancer.

[50] Alpízar-Alpízar W, Laerum OD, Christensen IJ (2016). Tissue inhibitor of Metalloproteinase-1 is confined to tumor-associated Myofibroblasts and is increased with progression in gastric adenocarcinoma. J Histochem Cytochem.

[51] Das AM, Koljenović S, Oude Ophuis CM (2016). Association of TIMP3 expression with vessel density, macrophage infiltration and prognosis in human malignant melanoma. Eur J Cancer.

[52] Hojilla CV, Wood GA, R l K (2008). Inflammation and breast cancer: metalloproteinases as common effectors of inflammation and extracellular matrix breakdown in breast cancer. Breast Cancer Res.

[53] Żekanowska E, Kotschy M, Rość D (1999). Blood concentration of plasmin-alpha 2 antiplasmin complexes in patients with non-small-cell lung cancer. Med Sci Monit.

[54] Yoneda K, Iida H, Endo H (2009). Identification of cystatin SN as a novel tumor marker for colorectal cancer. Int J Oncol.

[55] Placencio VR, DeClerck YA (2015). Plasminogen activator Inhibitor-1 in Cancer: rationale and insight for future therapeutic testing. Cancer Res.

[56] Poettler M, Unseld M, Mihaly-Bison J (2012). The urokinase receptor (CD87) represents a central mediator of growth factor-induced endothelial cell migration. Thromb Haemost.

[57] Bifulco K, Longanesi-Cattani I, Liguori E (2013). A urokinase receptor-derived peptide inhibiting VEGF-dependent directional migration and vascular sprouting. Mol Cancer Ther.

[58] Bianconi D, Unseld M, Prager GW (2016). Integrins in the Spotlight of Cancer. Int J Mol Sci.

[59] Rao JS, Gondi C, Chetty C (2005). Inhibition of invasion, angiogenesis, tumor growth, and metastasis by adenovirus-mediated transfer of antisense uPAR and MMP-9 in non-small cell lung cancer cells. Mol Cancer Ther.

