# Supplementary material for: Mature and progenitor endothelial cells perform angiogenesis also under protease inhibition: the amoeboid angiogenesis
Source: J Exp Clin Cancer Res. 2018 Apr 3;37:74. doi: 10.1186/s13046-018-0742-2 (PMC5883600; doi:10.1186/s13046-018-0742-2)
Supplement: Supplementary file 1 — Supplementary Materials and Methods. (DOCX 18 kb) [file 13046_2018_742_MOESM1_ESM.docx]

**Additional file 1**

**Materials and Methods**

**Time-lapse capillary morphogenesis** Briefly, cells were sparsely seeded on a 96 multiwell plate after a matrigel coating in the absence and in the presence of protease inhibitor mix. After an overnight incubation, time-lapse imaging of a field captured in the center of each plate was performed at an interval of 12,5 minutes for a total duration of about 5 hours. Images were captured by the time-lapse videomicroscopy using a Zeiss inverted phase-contrast microscope (Carl Zeiss Inc., Thornwood, NY) equipped with a 10X objective, Panasonic charge-coupled device cameras, and JVC BR9030 time-lapse video recorders.

**3D-invasion assay with Boyden chambers**

In order to increase uPAR dependency of the invasion process, the Matrigel was enriched with human vitronectin (VN) (Sigma-Aldrich, cat. V8379), to a final concentration of 300 μg/ml. To obtain a “thick” layer, we added 100 μl of Matrigel/cm^2^ porous membrane. According to the manufacturer’s instruction such an amount results into a 500 μm thick Matrigel layer, corresponding to about 30-50 folds the average diameter of ECs and ECFCs [1,2]. ECs and ECFCs (25 x 10^3^) were placed in the upper well of the Boyden chamber, and invasion was performed at 37 °C in 5% CO2 for 12 hours, a time consistent with the average speeds of cell migration under amoeboid and mesenchymal conditions in 500 μm thick gels [3]. The filters were removed and fixed in methanol. Non-invading cells on the upper surface of the filter were removed with a cotton swab, while invasive cells adherent on the lower filter surface were stained and counted using a light microscope (40x magnification) in 10 random fields/filter. Mobilization was measured by counting the number of cells moving across the filter. Each experiment was performed in triplicate and results were expressed as the absolute number of migrated cells ± SD or as % of control.

**Western Blotting**

Cells before and after specific treatments were lysed in a 10 mM Tris-HCl buffer, pH 7.4, containing 150 mM NaCl, 1% Triton X-100, 15% glycerol, 1mM sodium orthovanadate, 1 mM NaF, 1 mM EDTA, 1Mm phenylmethylsulphonyl fluoride and 10 µg aprotinin per 100ml. The same lysis buffer was used to solubilize beads-adsorbed active forms. Both beads and cell lysate proteins (40–100 µg) were subjected to 12% sodium dodecyl sulfate–polyacrylamide gel electrophoresis (SDS-PAGE) under reducing conditions and then blotted to a PVDF membrane (Hybond-C Extra; Amersham Biosciences) for 3 hours at 35V. The membrane was incubated with 5% skimmed milk in 20 mM Tris Buffer, pH 7.4, for 1 h at room temperature to block non-specific binding and then probed with primary antibody to RhoA and Rac1 (Mouse monoclonal 1:800 Millipore), uPAR (Rabbit polyclonal 1:400 FL290, Santa Cruz Biotechnology) and GAPDH (Rabbit policlonal 1:10000 Abcam), p-VEGFRII (Rabbit polyclonal 1:1000 Cell Signalling), KDR (Rabbit polyclonal 1:500 Millipore), pMLC2 and MLC (Rabbit polyclonal 1:1000 Cell Signalling), WAVE (Rabbit monoclonal 1:1000 Cell Signalling) overnight at 4° C. After incubation with horseradish peroxidase-conjugated donkey anti-mouse or anti-rabbit IgG (1:5000) for 1h (Amersham Bioscience, Rainham, UK), immune complexes were detected with enhanced chemiluminescence ECL detection system (Amersham Biosciences) for 1-30 min.

**Co-immunoprecipitation**

Protein concentration was determined using Bradford's method and 500 µg of total proteins were used. Integrin αvβ3 and interacting proteins were co-immunoprecipitated with anti-αvβ3 integrin antibody (Mouse monoclonal 1:500 Thermo Scientific) per 500 µg of total proteins, using dynabeads protein G (Novex, Life Technologies, Waltham, MA USA) according to the manufacturer’s instructions. Proteins bound to the beads were solubilized in Laemmli buffer, denatured to allow detachment of proteins from the beads, centrifuged and the supernatant separated by SDS–PAGE. After electro-blotting, nitrocellulose membranes were blocked and probed, overnight at 4°C, with the rabbit polyclonal anti-uPAR (Mouse monoclonal 1:400 Thermo Fisher) and the mouse monoclonal anti-αvβ3 integrin (Mouse monoclonal 1:500 Thermo Scientific). Then, the membranes were rinsed, incubated with peroxidase-conjugated anti-mouse or anti-rabbit immunoglobulin G (1 h, room temperature). After extensive washes, the reaction was revealed using the detection system from GE Healthcare (Milano, Italy the Super Signal West). A lysate that was treated with non-specific IgG (and Protein A/G), instead of the antibody, was used as negative control. The IgG should not bind any proteins in the sample then, after performing Western blot analysis, what we see at the level of the protein we are interested in, is a background that should be subtract from the control and the M25-treated samples.

**Immunofluorescence analysis**

Cells were grown on coverslips in their culture conditions. Once at confluence, cells were treated with physiological protease inhibitor cocktail and where required with scramble M25 and M25 peptide. After treatment cells were fixed in paraformaldehyde according to routine immunocytochemistry methods. The anti-human primary antibodies used were: uPAR (Mouse monoclonal 1:40 Thermo Fisher), KDR (Rabbit polyclonal 1:100 Millipore), anti-αvβ3 integrin (Mouse monoclonal 1:500 Thermo Scientific), anti-αvβ3 integrin (Mouse monoclonal 1:100 Thermo Scientific). The secondary antibodies used for single and double immunostainings were: CY3-conjugated antimouse IgG (1:800; C2181; Sigma-Aldrich) and FITC-conjugated anti-rabbit IgG (1:800; F-4151; Sigma-Aldrich). After the secondary antibody incubation, where was required, TRITC-labelled phalloidin (P1951, Sigma) was applied to the cells to visualize cell morphology and the arrangement of actin cytoskeleton. Nuclei were stained with the fluorescent Hoechst 33342 dye (DAPI) (10 µg/ml) (Invitrogen) for 15 min at RT. The coverslips containing the labelled cells were mounted with an anti-fade mounting medium (Biomeda, Foster City, CA) and observed under a Bio-Rad MRC 1024 ES Confocal Laser Scanning Microscope (Bio-Rad, Hercules, CA) equipped with a 15 mW Krypton/Argon laser source for fluorescence measurements. The cells were examined with a Nikon Plan Apo X60-oil immersion objective using an excitation wavelength appropriate for Alexa 488 (495 nm). Series of optical sections (XY: 512 x 512 pixels) were then taken through the depth of the cells with a thickness of 1 µm at intervals of 0.8 µm (Z step). A single composite image was obtained by superimposition of twenty optical sections for each sample observed. The collected images were analysed by ImageJ software.

**siRNA uPAR knock-down and quantitative Real-Time PCR analysis.**

Targeting and not-targeting siRNAs were obtained from Dharmacon (Carlo Erba Reagents, Milan, Italy). Specific silencing of uPAR gene was performed by transfection of ECFC and HMVEC with small-interfering-RNA (siGENOME SMARTpool), according to the manufactures's instructions. Not-targeting siRNA pool constructs were used as negative control (siCONTROL). To favour cell internalization siRNAs were incorporated into cationic liposomes, utilizing DharmaFECT transfection reagent. Cells were incubated with transfection mix (24–48 h for mRNA analysis and 48 h for protein and phenotypic analysis, respectively).

Total RNA was prepared using Nucleospin RNA II (Macherey-Nagel), agarose gel checked for integrity, and reverse transcribed with Go Script system (Promega) using random primers according to manufacturer's instructions. uPAR expression in ECFCs and HMVECs after uPAR siRNA-silencing, was determined by a quantitative Real-Time (RT)–PCR with an Applied Biosystem 7500 Fast Real Time PCR System (Applied Biosystems, Milano, Italy) and determined by the comparative Ct method using 18S ribosomal RNA as the normalization gene. Amplification was performed with the default PCR setting: 40 cycles of 95°C for 15 seconds and of 60°C for 60 seconds using SYBR Green–based detection (GoTaq qPCR Master Mix; Promega). Primers (IDT, Tema Ricerca, Italy) used for RT-PCR were as follows:

18S-rRNA: sense,5'-CCAGTAAGTGCGGGTCATAAG-3'; antisense, 5'-GCCTCACATAACC-ATCCAATC-3'; uPAR: sense, 5'- GCCCAATCCTGGAGCTTGA-3; antisense, 5'-TCCCCTTGC-AGCTGTAACACT-3’

References:

1. Christenson LK, Stouffer RL. Isolation and culture of microvascular endothelial cells from the primate corpus luteum. Biol Reprod. 1996;55:1397-404.
2. Asahara T, Kawamoto A, Masuda H. Concise review: Circulating endothelial progenitor cells for vascular medicine. Stem Cells. 2011;29:1650-5.
3. Friedl P. Prespecification and plasticity: shifting mechanisms of cell migration. Curr Opin Cell Biol .2004;16:14-23.
